# Mapping the neuroanatomical abnormalities in a phenotype of male compulsive rats

**DOI:** 10.1186/s12993-023-00221-y

**Published:** 2023-11-06

**Authors:** Elena Martín-González, Ángeles Prados-Pardo, Stephen J. Sawiak, Jeffrey W. Dalley, Daniel Padro, Pedro Ramos-Cabrer, Santiago Mora, Margarita Moreno-Montoya

**Affiliations:** 1 Department of Psychology and Health Research Centre (CEINSA), University of Almería, Carretera de Sacramento s/n, 04120 Almería, Spain; 2 Department of Physiology, Development and Neuroscience, University of Cambridge, Cambridge, UK; 3 Department of Psychology, University of Cambridge, Cambridge, UK; 4 Department of Psychiatry, University of Cambridge, Cambridge, UK; 5 Center for Cooperative Research in Biomaterials (CIC biomaGUNE), Basque Research and Technology Alliance (BRTA), Paseo Miramón 182, 20014 Donostia-San Sebastián, Spain; 6 Ikerbasque, Basque Foundation for Science, 48013 Bilbao, Spain; 7 Department of Neuroscience, University of Copenhagen Panum Institute, Copenhagen, Denmark

**Keywords:** Compulsive behavior, Schedule-Induced Polydipsia, Magnetic resonance imaging, Voxel-based morphometry, Cortico-striatal-thalamic-cortical circuit, White matter

## Abstract

Compulsivity is considered a transdiagnostic dimension in obsessive–compulsive and related disorders, characterized by heterogeneous cognitive and behavioral phenotypes associated with abnormalities in cortico-striatal-thalamic-cortical circuitry. The present study investigated the structural morphology of white and gray matter in rats selected for low- (LD) and high- (HD) compulsive drinking behavior on a schedule-induced polydipsia (SIP) task. Regional brain morphology was assessed using *ex-vivo* high-resolution magnetic resonance imaging (MRI). Voxel-based morphometry of segmented MRI images revealed larger white matter volumes in anterior commissure and corpus callosum of HD rats compared with LD rats. HD rats also showed significantly larger regional volumes of dorsolateral orbitofrontal cortex, striatum, amygdala, hippocampus, midbrain, sub-thalamic nucleus, and cerebellum. By contrast, the medial prefrontal cortex was significantly smaller in HD rats compared with LD rats with no significant group differences in whole brain, ventricular, or cerebrospinal fluid volumes. These findings show that limbic cortico-basal ganglia structures implicated in impulse control disorders are distinct in rats that are vulnerable to develop compulsive behavior. Such abnormalities may be relevant to the etiology of compulsive disorders in humans.

**Supplementary Information:**

The online version contains supplementary material available at 10.1186/s12993-023-00221-y.

Compulsions are repetitive, stereotyped thoughts and behaviors designed to reduce harm and are performed according to rigid rules [1]. Compulsive behaviors are driven by repetitive urges and typically involve the experience of limited voluntary control over these urges [2]. Compulsions are not only a central symptom of obsessive–compulsive disorder (OCD), the paradigmatic example of compulsivity [3], but are also present in other neuropsychopathological conditions such as schizophrenia, autism spectrum disorder (ASD), attention-deficit hyperactivity disorder (ADHD), and addiction [4–6]. Obsessive Compulsive and Related Disorders (OCRDs) [4] includes body dysmorphic, hoarding, hair-pulling, skin picking and olfactory reference disorders and hypochondriasis, all sharing compulsions as a cardinal characteristic [7].

Regarding compulsive symptomatology, there are different and heterogeneous cognitive and behavioral phenotypes, related to response inhibition, cognitive flexibility, planning (and goal-directed behavior), working memory, and error monitoring [6]. Behavioral and cognitive variability may be caused by distinct aberrant brain circuits centered on the ‘‘cortico-striatal loop’’ system [8, 9]. Magnetic Resonance Imaging (MRI) studies have demonstrated increased connectivity between Prefrontal Cortex (PFC) and striatum in OCD [10–12]. Thus, several studies reveal a dorsolateral Prefrontal Cortex (dlPFC)—striatum hypoactivity and a compensatory activation of Anterior Cingulate Cortex and ventrolateral Prefrontal Cortex (vlPFC) in non-medicated OCD [13] and first-degree OCD relatives [14]. Moreover, OCD patients show ventromedial Prefrontal Cortex (vmPFC) hypoactivity during a recall memory task [15] or during symptom provocation [16] and a lack of a safety signal computed by this structure [17]. There is also a relationship between the OFC and the striatum in OCD patients confirmed by meta-analyses of a variety of neuroimaging studies [18]. Moreover, there exists a hyperactivity of the lateral OFC in OCD patients during symptom provocation normalized over the course of behavioral therapy [19]. Finally, this frontostriatal dysregulation present in OCD patients is normalized by Deep Brain Stimulation (DBS) in the ventral striatum and transcranial magnetic stimulation in the mPFC [10, 20].

However, inhibitory control deficit seems to be driven by different brain areas and its aberrant connectivity with the cortico-striatal system. For instance, inputs to the striatum are relayed from midbrain neurons in Ventral Tegmental Area (VTA) and Substantia Nigra (SN) [21–23]. Plasticity mechanisms in these areas are implicated in habit formation [24] and in a multitude of pathological conditions, including OCD, ADHD, Parkinson disease, Huntington disease, Tourette syndrome, and schizophrenia [25]. Hippocampus and amygdala are postulated to play a central role in the neurobiology of OCD through mediation of cognitive and affective processes. Volumetric abnormalities in hippocampus [26–30], its subregions [31–33], and amygdala [34–37] are reported in disorders with compulsive symptomatology. Furthermore, neuromodulation intervention of OCD points to several areas that may also be involved in the expression of compulsive symptoms: Presupplementary Motor Area (PSMA) and Supplementary Motor Area (SMA) are the most promising brain regions for Transcranial Direct Current Stimulation (tDCS) [38–42] and Subthalamic Nucleus (STN) seems to be an effective target for DBS [43–45]. Finally, increasing evidence reveals the cerebellum as an important structure of fronto-striatal circuit [46–49], highlighting its important role in higher-order cognitive functions [50, 51]. Clinical studies have found that the connectivity between cerebellum and PFC is lower, while connectivity with basal ganglia is stronger in OCD patients [14] suggesting less top-down control over the PFC on the lower regions.

Schedule-induced polydipsia (SIP) procedure is characterized by the development of an adjunctive behavior of excessive drinking in food-deprived animals exposed to intermittent food-reinforcement schedules [52, 53]. Translationally, psychogenic polydipsia is a similar phenomenon characterized by compulsive non-regulatory fluid consumption present in > 20% of chronic psychiatric patients, that has been linked compulsive spectrum disorders [54–57]. As drinking behavior on SIP is an excessive, persistent, and maladaptive behavior, SIP is one of the most well-established preclinical models for the study of neuropsychopathological disorders presenting compulsive behavior such as OCD, schizophrenia and alcohol abuse [58–62]. Thus, SIP seems to meet the criteria as a valid model of compulsive behavior [60]. Moreover, different studies have demonstrated relevant individual differences in SIP acquisition [63–65]. Indeed, two populations can be selected according to their SIP acquisition: High Drinker (HD) rats, considered as compulsive, versus Low Drinker (LD) rats, considered as non-compulsive [60]. SIP preclinical model allows us to identify a compulsive vulnerable population to study the brain correlates underlying compulsive spectrum disorders due to their transdiagnostic profile [60, 66].

The aim of the present study was to investigate the morphology of brain differences in white and gray matter structures in the compulsive phenotype of rats selected by SIP using high-resolution magnetic resonance imaging, in order to clarify the neuroanatomical substrates related to OCRDs.

## Methods and materials

### Animals

Twenty-four male Wistar rats from Envigo (Barcelona, Spain) were used in the present study. The animals were housed in social groups of four per cage, kept in a temperature-controlled environment at 22 °C, and with a 12:12 h light–dark cycle. Water and food were freely available and environmental enrichment was provided throughout the experiment. After 10 days for habituation animals through controlled feeding were gradually reduced to 85% of their free-feeding body weight. All procedures were conducted in accordance with the Spanish Royal Decree 53/2013 and the European Community Directive (2010/63/EU) for animal research. The present study was also approved by the Animal Research Committee from the University of Almería and complied with the ARRIVE guidelines (Additional file 1).

### SIP procedure

Animals were tested in 8 standard operant chambers (32 × 25 × 34 cm) (MED Associates, St. Albans, VT, USA) equipped with a bottle of water, pellet dispenser and ambient light. Animals were exposed to a food pellet presentation using a fixed time 60 s (FT-60 s) schedule during 60 min sessions with free access to a bottle of tap water. Following the protocol described in [60] and after the 20 daily sessions, rats were divided into low drinkers (LD) and high drinkers (HD), depending on whether their water consumption (average of the last 5 sessions) was above or below the median of the group. Amount of water consumed (milliliters), total number of licks in the bottle, and total number of magazine entries were registered [67] (Additional file 1).

### Cerebral MRI volumetric assessment

Immediately after the last SIP session and the separation into HD and LD rats, animals were perfused with 4% PFA, and the whole skull was stored in PFA prior to high-resolution ex-vivo analyses in the University of Cambridge. Brains were scanned intact inside the cranium using MRI at 9.4 Tesla using a Bruker BioSpec 94/20 system with the manufacturer-provided 4-channel rat brain array coil with an 86 mm birdcage transmit/receive coil [68]. (Parameters in Additional file 1).

### Cerebral MR image processing

The user bias-free automatic pipe-line for image processing included the following steps: Images were treated for bias field correction using the ITK implementation of the N4 algorithm in python [69]. Segmentation of the brain and removal of signal from skull and external tissues was achieved by Brain extraction using the rBET software [70]. A normalization algorithm was implemented in Python to normalize signal intensities from different scans. Finally, each individual brain image was co-registered to a common space using the SIGMA rat brain atlas for reference [71]. For this task we used ANTs, the ANTsX ecosystem for quantitative biological and medical imaging [72]. Segmented regions of interest (ROIs) of the brain atlas were used to calculate volumes and signal intensities for those regions for each individual brain (Additional file 1).

### Data analysis

SIP acquisition was analyzed using a two-way repeated-measures analysis of variance (ANOVA), with “group” (LD and HD) as between-subject factor and “sessions” (20 sessions) as the within-subject factor. The differences between groups in the volume of the different cerebral areas were studied using Student’s t-test (T-test). When appropriate, post hoc analyses were performed using Bonferroni correction. Statistical significance was established at p < 0.05. Effect size was reported when appropriate. All analyses were performed using Statistica® software (version 8.0) and all figures were made using GraphPad Prism 8 (Additional file 1).

## Results

### Screening for compulsivity on the schedule-induced polydipsia task

**Fig. 1:**
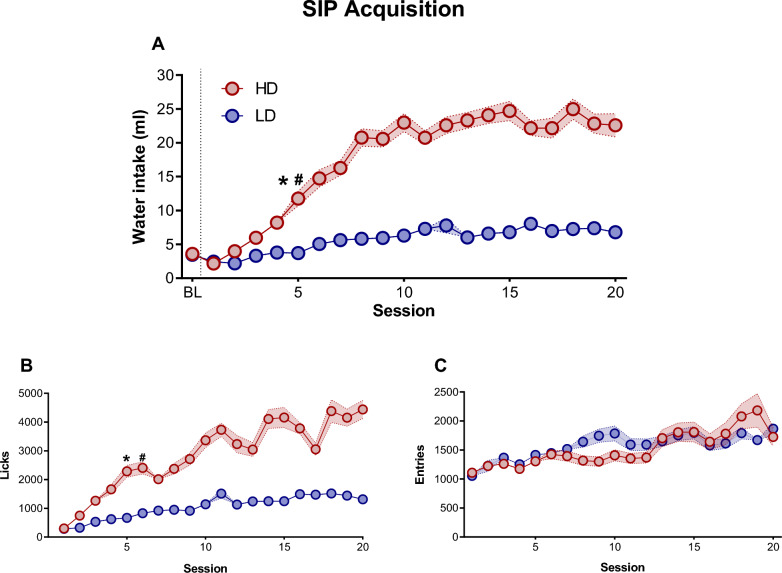
Schedule-Induced Polydipsia. The mean (± SEM) water intake (**A**), total number of licks (**B**), and magazine entries (**C**) in FT-60 s across 20 sessions of Schedule-Induced Polydipsia (SIP) in High drinker (HD, n = 12) and Low drinker (LD, n = 12) rats. *p < 0 .05 indicates significant differences between HD and LD rats from that session onward. ^#^p < 0 .05 indicates significant differences from that session onward compared with session 1 in the same group

The mean water intake, total licks and total magazine entries in LD and HD over 20 SIP sessions are shown in Fig. 1. Concerning the water intake, repeated measures ANOVA revealed significant differences according to the interaction between the SIP acquisition sessions and LD vs HD (interaction SIP session × group effect: F(19,418) = 14,89, p < 0.001; η^2^_p_ = 0.4). Repeated measures ANOVA and η2p also showed a significant interaction in total number of licks (interaction SIP session × group effect: F(19, 418) = 5.94, p < 0.001; η^2^_p_ = 0.21). Post hoc analysis indicated that SIP induced different rates in drinking behavior across the 20 sessions in both groups. In water intake, the LD and HD groups differed in session 5 (p < 0.001; d = 1.65) and the HD group increased their water consumption in session 5 (p < 0.001; d = 2.01) compared to session 1. Similar differences between LD and HD were found in total number of licks: the LD and HD group differed in session 5 (p < 0.01; d = 1.68) and the HD group increased their number of licks in session 5 (p < 0.001; d = 2.18) compared to session 1. There were no significant differences between LD and HD animals in the total magazine entries on SIP (SIP session interaction × group effect: F(19, 418) = 1.23, p = 0.23). Please, note that the effect showed on SIP between HD and LD groups might not be due to a difference in motivation or reward processing, as both groups did not show differences in magazine entries. Therefore, these differences are associated to the performance of excessive and persistent drinking behavior, measured by water consumed and licking behavior on SIP (For a review see [60, 73, 74].

### Cerebral MRI volumetric assessment

The following subsections show the significant brain volumetric differences in percentage (in relation to total brain volume) between HD and LD rats assessed by MRI and their relationship with SIP. The results are organized into: (1) general measures (whole brain volume, WM, GM, and CSF); (2) WM areas; (3) GM cortical areas; and (4) GM subcortical areas from anterior to posterior according to the Paxinos and Watson [75] brain atlas. In supplementary information: brain volumetric results in mm^3^ and no significant differences in Additional file 1: Table S1, and correlations between SIP variables and volumetric measures are presented in Additional file 1: Table S2 and S3.

#### Whole brain gray matter, white matter, and cerebrospinal fluid

**Fig. 2:**
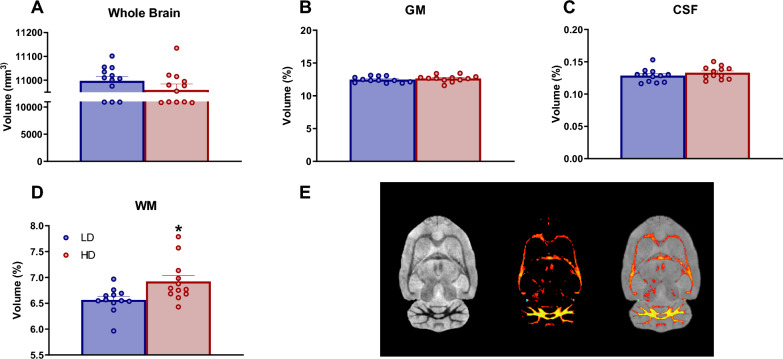
Volumetric MRI data of whole brain (**A**), GM (**B**), CFS (**C**) and WM (**D**). Scheme of brain segmentation (**E**). Data are expressed as the means ± SEM. *p < 0.05 indicates significant differences between LD and HD rats. CSF: cerebrospinal fluid; GM: gray matter; WM: white matter

The percentage of volume of whole brain, gray matter (GM), white matter (WM), and cerebrospinal fluid (CSF) are shown in Fig. 2. No significant differences between groups were observed in whole brain volume (Fig. 2A; total volume in mm^3^: df = 22; T-test = 1.19; p = 0.24), GM (Fig. 2B; df = 22; T-test = − 0.93; p = 0.36) or CSF (Fig. 2C; df = 22; T-test = − 1.11; p = 0.28). However, T-test analysis revealed an increased percentage of WM volume in HD animals compared to LD animals (Fig. 2D.; df = 22; T-test = − 2.66; p < 0.05; d = 1.09).

#### White matter structures

**Fig. 3:**
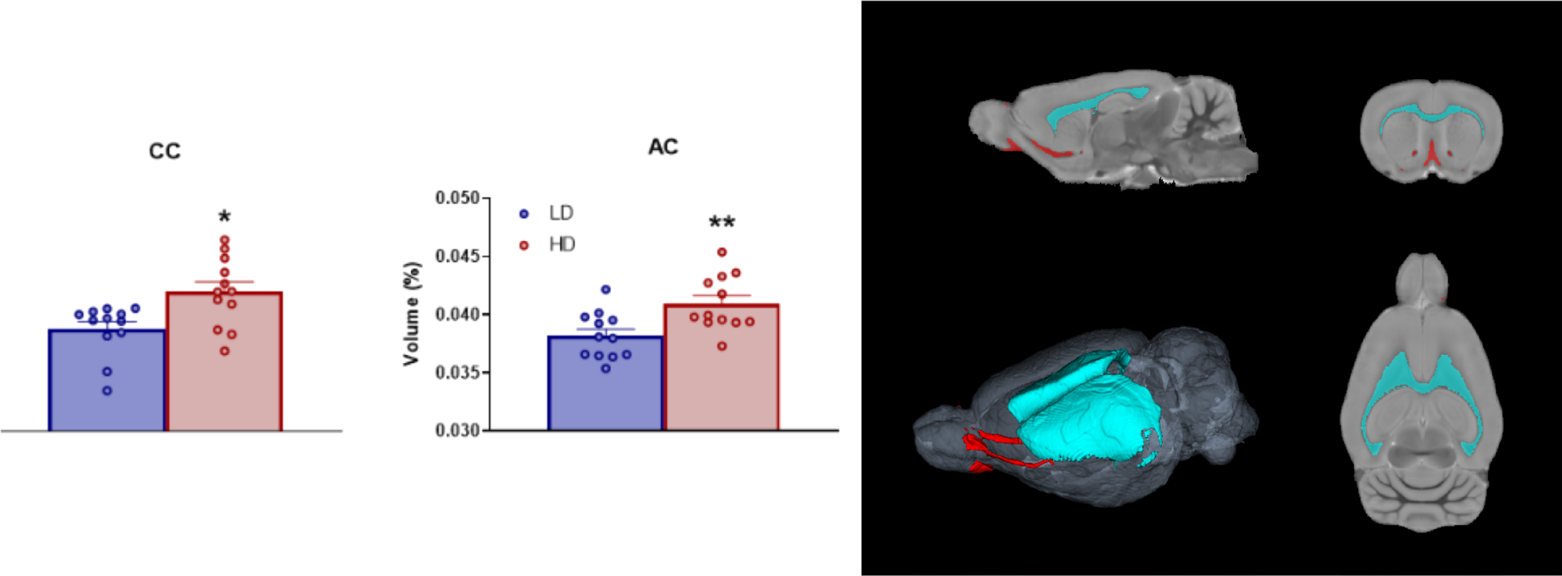
Volumetric MRI data of selected brain white matter structures. (Left). Statistical differences between groups in corpus callosum (CC), and Anterior Commissure (AC). (Right) One sagittal plane (top left), one transverse plane (top right), one coronal plane (bottom right) and a 3D rendered representation (bottom left) of the selected regions of interest analyzed including CC in cyan and AC in red. Data are expressed as the means ± SEM. *p < 0.05; **p < 0.01 indicate significant differences between LD and HD rats. (note: for some 2D views is not possible to visualize all ROIs in a single plain)

Volume in percentage of WM areas with statistical differences are shown in Fig. 3. T-test analysis revealed that HD animals showed an increased volume in the Corpus Callosum (CC) (Fig. 3B; df = 22; T-test = − 2.95; p < 0.05; d = 1.4) and Anterior Commissure (AC) (Fig. 3C; df = 22; T-test = − 3.1; p < 0.01; d = 1.38) compared to LD animals.

#### Gray matter structures: cortical areas

**Fig. 4:**
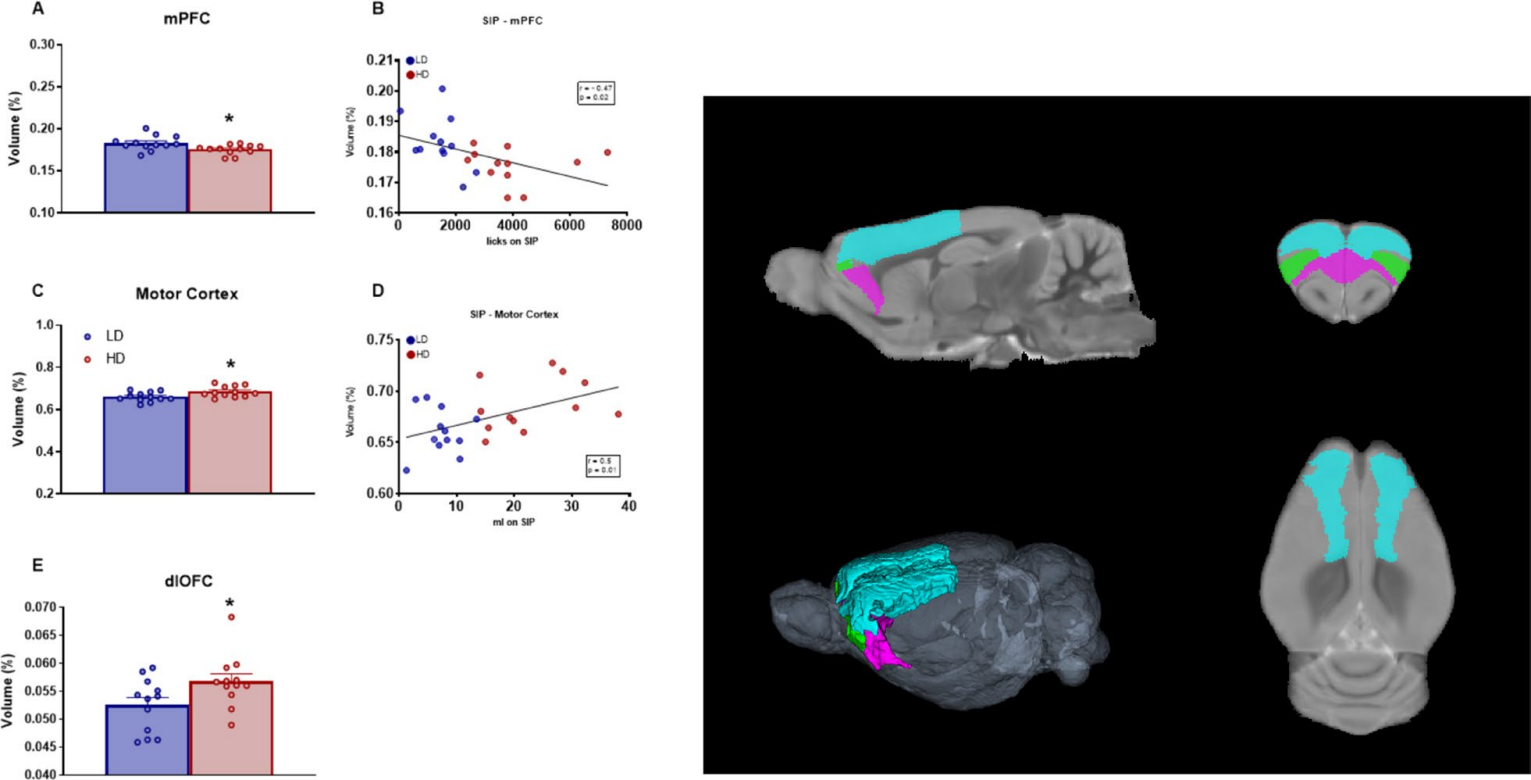
Volumetric MRI data of selected prefrontal brain grain matter structures. (Left). Statistical differences between groups in **A**, **B** medial prefrontral cortex (mPF), **C**, **D** Motor Cortex (MC), and **E** dorsolateral orbitofrontal cortex (dlOFC). (Right) One sagittal plane (top left), one transverse plane (top right), one coronal plane (bottom right) and a 3D rendered representation (bottom left) of the selected regions of interest analyzed including mPFC in magenta, MC in cyan and dlOFC in green. Data are expressed as the means ± SEM. *p < 0.05; **p < 0.01 indicate significant differences between LD and HD rats. (note: for some 2D views is not possible to visualize all ROIs in a single plain)

Volume in percentage of GM cortical areas with statistical differences between groups are shown in Fig. 4. T-test analysis revealed that HD animals showed an increased volume of motor cortex (Fig. 4C; df = 22; T-test = − 2.72; p < 0.05; d = 1) and dlOFC (Fig. 4E; df = 22; T-test = − 2.19; p < 0.05; d = 0.85) compared to LD animals. However, compulsive HD presented a decreased volume of mPFC compared to LD rats (Fig. 4A; df = 22; T-test = 2.54; p < 0.05; d = 1,13).

Water consumed (ml) during the last 5 sessions on SIP correlated with volume of motor cortex (Fig. 4D; r = 0.5; p < 0.05). Moreover, licking behavior during the last 5 sessions on SIP correlated with volume of Motor Cortex (r = 0.57; p < 0.01) and mPFC (Fig. 4B; r = − 0.47; p < 0.05).

#### Gray matter structures: subcortical anterior areas

**Fig. 5:**
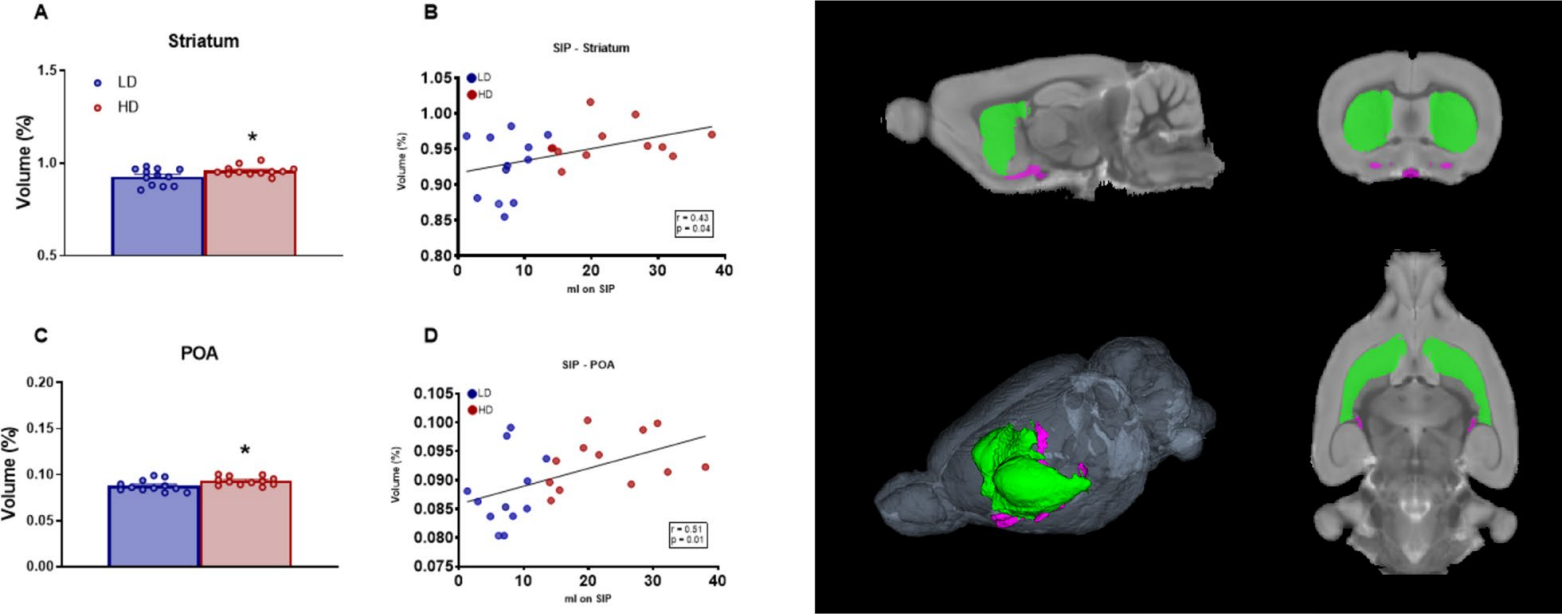
Volumetric MRI data of selected brain gray matter structures. **(**Left) Statistical differences between groups in **A**, **B**) Striatum (ST), and **C**, **D** Preoptic area (POA). (Right) One sagittal plane (top left), one transverse plane (top right), one coronal plane (bottom right) and a 3D rendered representation (bottom left) of the selected regions of interest analyzed including Striatum in green and POA in purple. Data are expressed as the means ± SEM. *p < 0.05 indicates significant differences between LD and HD rats

Volume in percentage of GM subcortical anterior areas with statistical differences between groups are shown in Fig. 5. T-test analysis revealed that HD animals showed an increased volume in striatum (Fig. 5A; df = 22; T-test = − 2.44; p < 0.05; d = 1.26), and Preoptic Area (POA) (Fig. 5C; df = 22; T-test = − 2.59; p < 0.05; d = 1.17) compared LD rats.

Moreover, water consumed (ml) during the last 5 sessions on SIP correlated with volume of Striatum (Fig. 5B; r = 0.43; p < 0.05) and POA (Fig. 5D; r = 0.51; p < 0.01).

#### Gray matter structures: subcortical medial areas

**Fig. 6:**
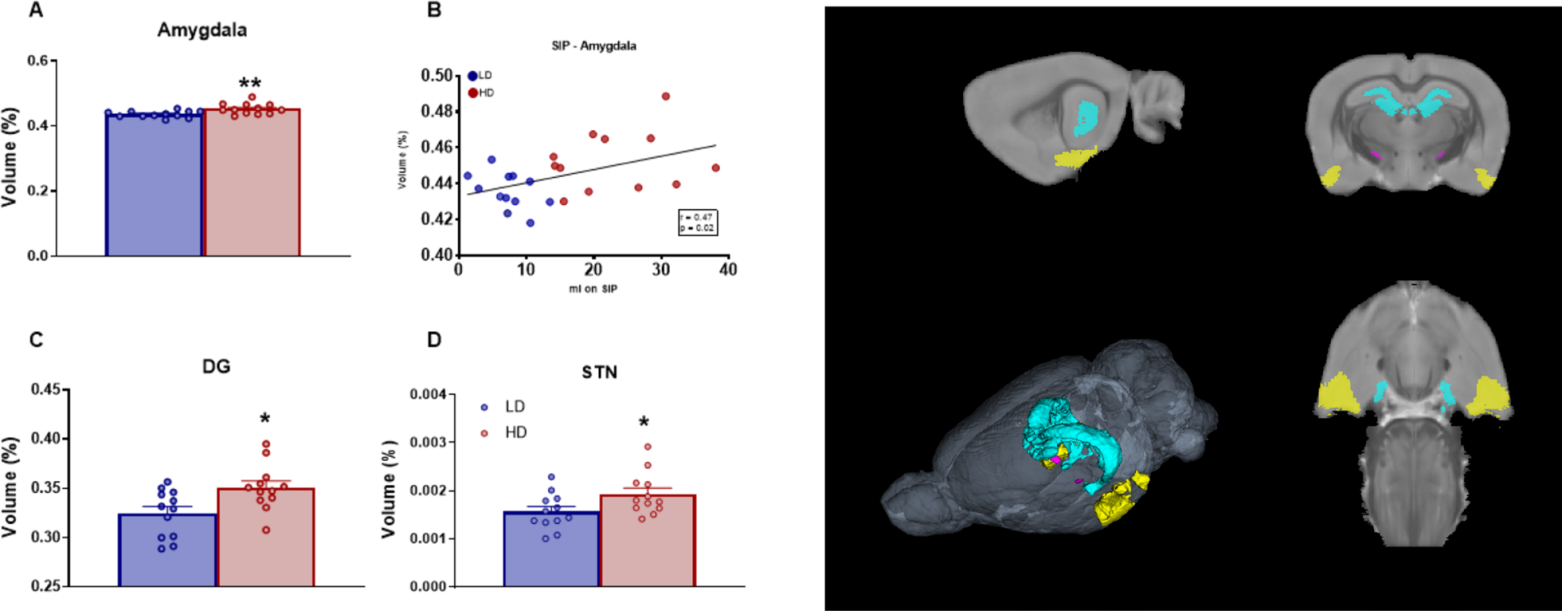
Volumetric MRI data of subcortical medial gray matter structures. (Left) Statistical differences between groups in **A**, **B** Amygdala, **C** Dentate gyrus (DG), and **D** Subthalamic nucleus (STN). (Right) One sagittal plane (top left), one transverse plane (top right), one coronal plane (bottom right) and a 3D rendered representation (bottom left) of the selected regions of interest analyzed including: Amygdala in yellows, DG in cyan and STN in purple. Data are expressed as the means ± SEM. *p < 0.05; **p < 0.01 indicate significant differences between LD and HD rats. (for some 2D views is not possible to visualize all ROIs in a single plain)

Volume in percentage of GM subcortical medial areas with statistical differences between groups are shown in Fig. 6. T-test analysis revealed that HD animals showed increased volume in amygdala (Fig. 6A; df = 22; T-test = − 3.21; p < 0.01; d = 1.54), dentate gyrus (DG) (Fig. 6C; df = 22; T-test = − 2.72; p < 0.05; d = 1.5) and STN (Fig. 6D; df = 22; T-test = − 2.18; p < 0.05; d = 0.91).

Water consumed (ml) during the last 5 sessions on SIP correlated with volume of Amygdala (Fig. 6B; r = 0.47; p < 0.05).

#### Gray matter structures: subcortical posterior areas

**Fig. 7:**
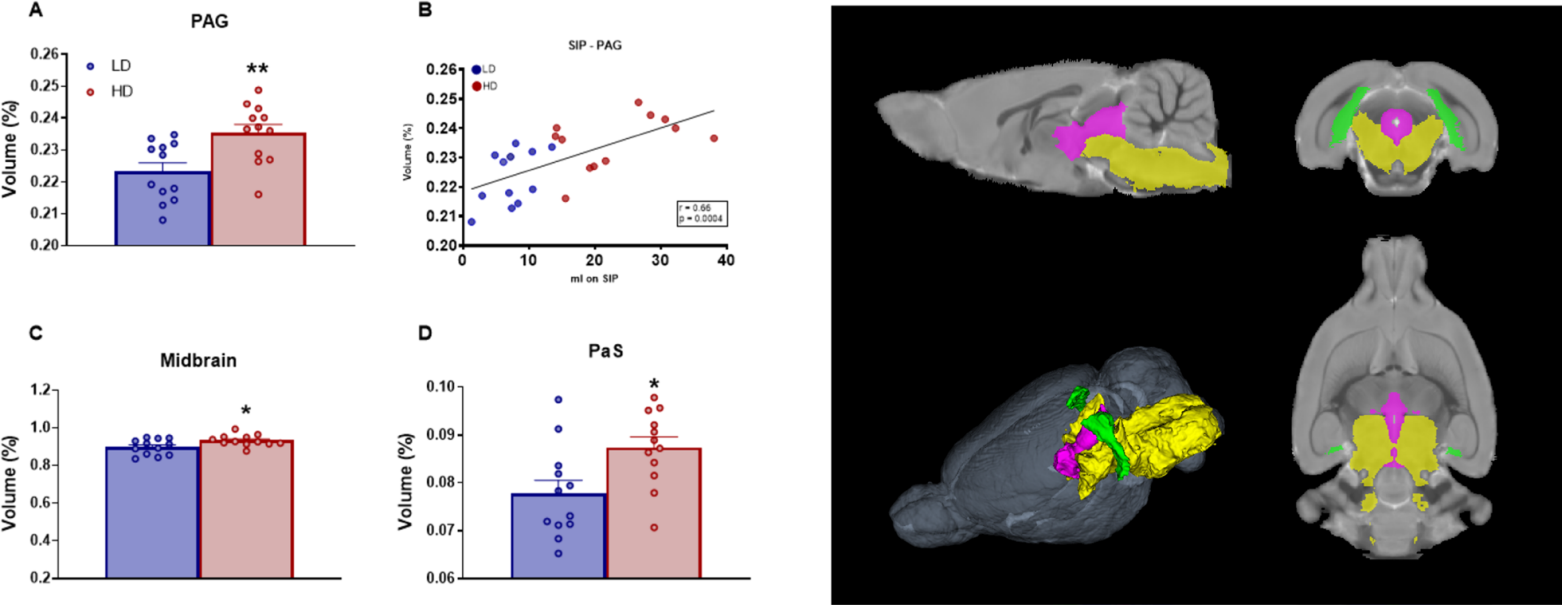
Volumetric MRI data of subcortical posterior gray matter structures. (Left) Statistical differences between groups in **A**, **B** Periaqueductal gray matter (PAG), **C** midbrain, and **D** parasubiculum (PaS). (Right) One sagittal plane (top left), one transverse plane (top right), one coronal plane (bottom right) and a 3D rendered representation (bottom left) of the selected regions of interest analyzed including: PAG is in green, midbrain in yellow and PaS in purple. Data are expressed as the means ± SEM. *p < 0.05; **p < 0.01 indicate significant differences between LD and HD rats. (note: for some 2D views is not possible to visualize all ROIs in a single plain)

**Fig. 8:**
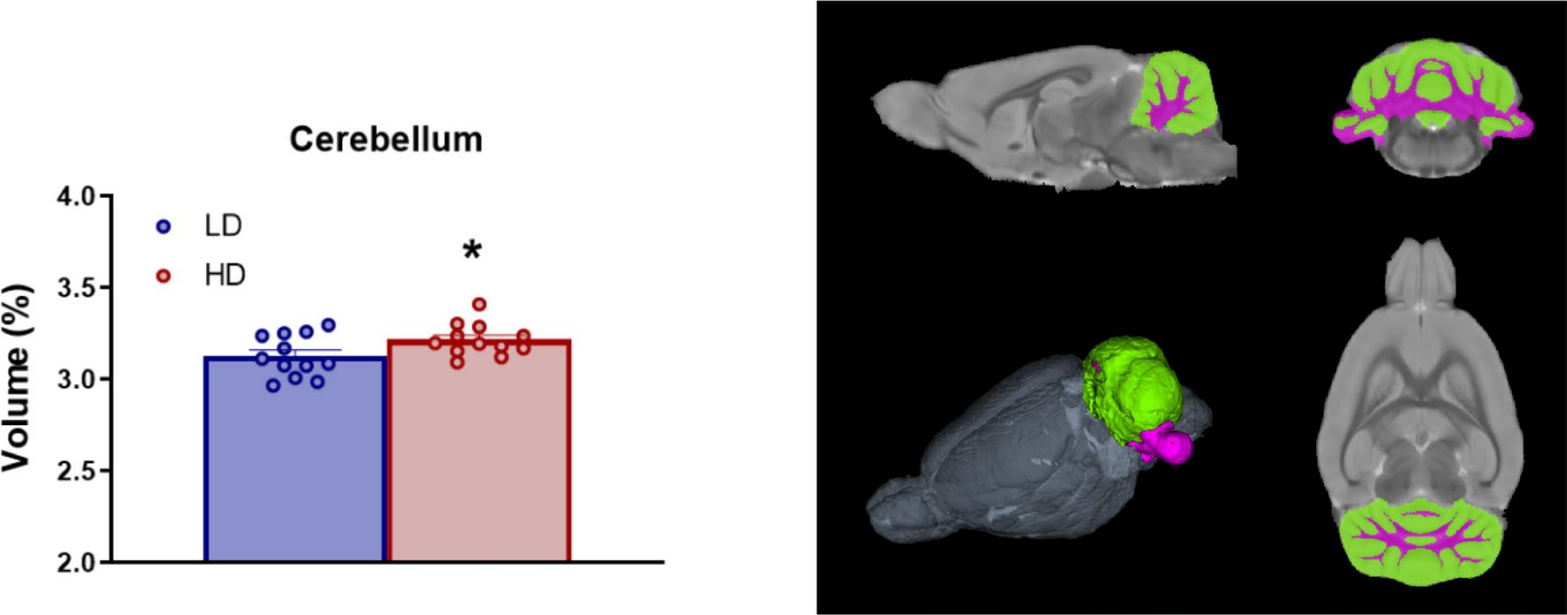
Volumetric MRI data of Cerebellum. (Left) Statistical differences between groups. (Right) One sagittal plane (top left), one transverse plane (top right), one coronal plane (bottom right) and a 3D rendered representation (bottom left) of the selected regions of interest analyzed including: Gray matter in green and white matter tracks in purple. Data are expressed as the means ± SEM. *p < 0.05; **p < 0.01 indicate significant differences between LD and HD rats

Volume in percentage of GM subcortical posterior areas with statistical differences between groups are shown in Fig. 8. T-test analysis revealed that HD animals showed increased volume in periaqueductal gray (PAG) (Fig. 7A; df = 22; T-test = − 3.2; p < 0.01; d = 2.22), midbrain (Fig. 7C; df = 22; T-test = − 2.46; p < 0.05; d = 0.85) and parasubiculum (PaS) (Fig. 7D; df = 22; T-test = − 2.68; p < 0.05; d = 1.13).

Water consumed (ml) during the last 5 sessions on SIP correlated with volume of PAG (Fig. 7B; r = 0.66; p < 0.001). Moreover, licking behavior during the last 5 sessions on SIP correlated with volume of PAG (r = 0.62; p < 0.001).

#### Cerebellum

Volume in percentage of cerebellum is shown in Fig. 8. T-test analysis revealed that HD animals showed increased volume in Cerebellum compared to LD animals (Fig. 8B; df = 22; T-test = − 2.37; p < 0.05; d = 0.99).

## Discussion

The present study explored the possible alterations of the morphology in different brain areas on a compulsive phenotype of rats selected by SIP. The neuroimaging assessment has considered the whole-brain, the cortico-striatal-thalamic-cortical pathway, as well as the associated neurocircuitry that involves the limbic and the cerebellar network. Voxel-based morphometry revealed that compulsive HD rats showed a significantly increased volume of white matter structures (CC and AC), cortical structures (motor cortex and dl OFC), subcortical structures (striatum, amygdala, DG, STN, PAG, and midbrain) and cerebellum relative to LD animals. However, HD rats showed a decreased volume of mPFC compared to LD rats. No differences were observed between HD and LD groups either in the whole brain or in cerebrospinal fluid (CSF) volume. These results highlight and extend the knowledge about brain morphological alterations in the compulsive phenotype, which may underlie the behavioral inhibition deficits observed.

### Compulsivity and structural brain assessment: white matter structures

Compulsive HD rats showed an increased general WM volume and its related structures, such as CC and AC compared to LD rats. Different preclinical studies on inhibitory control deficit have also revealed WM alterations. An abnormal increase of WM maturation was observed in an adolescent model of compulsive checking behavior [76], in selectively bred ASD/ADHD-like behavior rats [77], and in animals with repetitive traumatic brain injury (TBI) that showed impulsivity [78]. In line with our result, some studies have shown an increase in CC in an adolescent model of compulsive checking behavior [76], in selectively bred ASD/ADHD-like behavior rats [77], and in a female rat model of Fragile X syndrome characterized by autistic behaviors [79]. Moreover, OCD-like behavior mice exhibited increased c-fos expression in the AC ([80]. In contrast, in a previous study in our laboratory we found that HD rats selected by SIP showed reduced myelin basic protein (MBP) in the CC [61], as well as in a preclinical model of ASD [81, 82].

In line with our current findings, abnormal WM and myelin development have been proposed that may underlie several neuropsychiatric disorders [83]. Clinical studies using MRI observed increased WM in patients with OCD [84] and ASD [85–87], and WM volume was positively correlated with the severity of ritualistic/compulsive behaviors in adults and adolescents with anorexia nervosa [88]. However, the relationship between CC and AC and compulsive symptomatology is unclear. An increased CC volume has been linked to doubt-checking subclinical OC symptoms in healthy children [89], in ASD [90] and in pediatric OCD patients [91]. Moreover, the stereotaxic coordinates for DBS treatment for OCD are close to the AC [92]. However, a decreased volume of CC has also been associated with pediatric OCD [93], adult OCD [94], and ASD children [95].

### Compulsivity and structural brain assessment: cortico-striatal circuit

The neurocircuitry traditionally involved in habit learning and compulsive behaviors includes the striatum and its connections with frontal cortex regions [24, 96–98].

HD compulsive animals showed increased volume of striatum and dlOFC, but reduced volume of mPFC compared to LD rats. In accordance to our findings, a previous study showed that SIP acquisition in rats induced structural plasticity changes by an increase in dendritic spine density in dorsolateral striatum compared to control rats exposed to a mass feeding condition [99]. Moreover, previous studies on SIP have revealed an alteration in the OFC, such as increased c-fos activity in the lOFC in rats with SIP acquisition [58] and in high compulsive rats selected by SIP [100]. Although our result in mPFC contrasts with previous data in our laboratory, where no differences were observed in the PrL cortex and IL cortex volume between HD and LD rats [101], different studies have shown a reduction in mPFC volume in RHA animals characterized by impulsive and compulsive behaviors [102], and in a model of ADHD, the juvenile SHRs rats [103].

In clinical studies, comparable structural abnormalities in these brain areas have been reported. Neuroimaging studies showed increased GM volumes of striatum and its subregions in OCD [104, 105] and in ASD [106, 107]. Striatum volume also showed a positive association with compulsivity scores in subclinical adolescent population [108] and with the severity of restricted and repetitive behaviors in ASD [109]. Moreover, clinical studies in OCD patients have also shown an increased volume of OFC [104]. Finally, in accordance with our findings, some clinical studies have also reported a reduction of mPFC in inhibitory control disorders such as in subjects with online game addiction [110] and in individuals with heavy drinking profile [111]. Indeed, symptom improvement in OCD patients by the cognitive-behavioral therapy correlated with larger volume within the right mPFC [112].

### Compulsivity and structural brain assessment: cortico-striatal-thalamic-cortical circuit

In the assessment of the brain neurocircuitry implicated in compulsive behaviors, many authors also consider an extended network that involves other midbrain, thalamic and cortical areas [113].

In this sense, HD animals also presented an increased volume of motor cortex, STN and midbrain. As far as we know, motor cortex volume has not been fully studied in animal models of inhibitory control deficit. However, the different subregions of the motor cortex might have an encompassing role with the cortico-striatal network in a motor inhibition task [114] and in learning of simple sequences [115]. Moreover, when drug seeking is well established, it is under the dominant control of the dorsolateral striatum, which receives its major cortical afferents from the motor cortex [116]. Regarding the role of STN on compulsive behavior, stimulation or inactivation of STN have revealed to ameliorate the inhibitory control deficit in animal models of OCD [117, 118], of compulsive heroin taking [119], and of risk-preferring [120]. Finally, data similar to ours have been found in areas that compose the midbrain, such as increased volume of VTA in models of stress as maternally deprived animals [121], and correlation between maintained drug use despite negative consequences with PAG volume in a rat model of cocaine addiction [122].

Related to clinical studies, OCD patients had greater activation of the SMA during high- vs low-conflict trials in the multi-source interference Task [123] and a disruption in higher-order motor networks has been found in compulsive behavior such as skin-picking symptoms [124]. Moreover, in the clinical context, the bilateral DBS in the STN is a recommended treatment for refractory OCD [125]. Finally, an increase of midbrain [126] have been shown in OCD patients.

### Compulsivity and structural brain assessment: the role of limbic and cerebellar areas

Moreover, other relevant brain structures of the limbic network associated with compulsivity are hippocampus and amygdala. The present study found increased volume of the DG of the hippocampus and amygdala in HD rats compared to LD rats. Our data in DG contrasts with previous findings in our lab, where HD group had a reduced dorsal hippocampus volume compared to LD group measured by stereology [101]. However, a classical study showed that hippocampal lesions were followed by a rapid and stable SIP acquisition [127]. Regarding increased amygdala, similar data was found in HD animals [101] and in the high-avoidance Hatano rats [128] that showed increased BLA volume.

Clinical studies have linked hippocampal and amygdalar abnormalities to compulsive symptomatology. An increased volume of hippocampus have been found in OCD patients [33, 104] and in internet GD patients, where the hippocampus volume correlated with symptom severity [129]. Moreover, the association between different subregions of amygdala and compulsive trait have been found in OCD [130, 131], in a sub-clinical population [34], in subjects with compulsive sexual behavior [36] and in individuals with internet GD [129].

In the present experiment, HD animals showed an increase in the volume of cerebellum, which is in line with different cerebellar alterations found in animal models of ASD/ADHD-like behaviors [77] of autism [132], of addiction [133], and in animals with repetitive jumping behavior [134].

In clinical studies, according with our results, a higher volume of different areas of cerebellum was found in ASD [106, 135, 136] and in OCD patients [104, 137–139]. Interestingly, cerebellar volume correlated with OC symptom severity in OCD patient [105] and with emotional dysregulation severity in ADHD patients [140].

In summary, our findings reveal a collection of morphological abnormalities implicated in the compulsive phenotype selected by SIP, that suggest a brain network that includes the traditional cortico-striatal-thalamic-cortical circuit and other less studied brain areas of the limbic, and cerebellar circuit, which expand the knowledge about brain areas that might be implicated in inhibitory control. The increased volume of several areas observed might not be attributable to a possible water increment in the brain, because no significant differences were found in the whole brain, ventricles, or CSF volume between groups. Possibly, specific and dissociable circuits within the compulsivity brain network might be associated with different dysfunctions, highlighting the heterogeneity of the plausible endophenotypes of OCD [141].

However, our study presents certain limitations. The volumetric assessment of the brain areas is a powerful analysis tool to identify abnormalities in the morphological functioning of neurocircuits, but the current study is unable to determine the underlying mechanisms of the morphological differences observed. Presumably, the volumetric changes found suggest a possible aberrant plasticity in these brain areas linked to compulsive behavior. In this regard, it is known that variations in the volume of particular brain regions may reflect microscopic alterations including changes in synaptogenesis, dendritic arborization, number of neurites, and neuronal and glial genesis, that might in turn, influence behavioral responses [142–144]. Moreover, we have observed morphologic changes in large areas that includes a great diversity in their functional specialization according to each of its substructures. Further understanding of these alterations is necessary for future experiments, which must also be provided with female rats. Another limitation of our study is the discrepancy between the findings in preclinical studies, which might be attributable to the wide variety of models used. This reinforces the translational validity of the neuroimaging studies, since in the clinical literature, this lack of concordance is also found, which might be due to heterogeneity within neurodevelopmental disorders, comorbidity, age onset and effect of psychopharmacology treatments.

The development of compulsive drinking by SIP exposure might induce microstructural abnormalities in the cortico-striatal-thalamic-cortical circuit as well as in limbic and cerebellar areas in HD compulsive rats. These results suggest that SIP might potentially have a time-dependent role in modulating the brain plasticity, specifically in high compulsive vulnerable rats, the HD group selected by SIP. This hypothesis is in consonance with previous data in our laboratory where a brain volumetric assessment did not reveal significant differences between HD and LD rats in basal conditions, but the re-exposure to SIP induced significant changes only in HD animals [101]. Finally, this pattern is also found in the clinical literature, when the potential brain differences in compulsive patients become evident during the exposition to the problem situation [15, 16, 19], supporting SIP as a valid and translational model for the study of compulsivity.

## Conclusions

The present MRI study reveals a collection of morphological abnormalities and suggests the implication of frontostriatal circuit and its modulators, which might have different functions linked to compulsive behavior on SIP. HD animals presented increased general WM volume compared to LD animals without differences in GM or CSF volume. HD rats also showed increased volume in white matter structures such as CC and AC. Altered volume of cortical areas were found in HD rats: decreased volume in mPFC and increased volume of Motor Cortex and dlOFC. Moreover, subcortical areas have been increased in HD phenotype: striatum, DG, amygdala, midbrain, PAG and STN. This pattern of alterations might be related to a vulnerability to develop compulsive behavior, which might be exacerbated by SIP exposure, and point toward SIP as a suitable preclinical model for enhancing the knowledge about the vulnerability to OCRDs.

### Supplementary Information

**Additional file 1.** Supplementary Information.

## Data Availability

The data supporting the findings of this study are available from the corresponding author upon reasonable request.
