# Supplementary material for: Mapping the neuroanatomical abnormalities in a phenotype of male compulsive rats
Source: Behav Brain Funct. 2023 Nov 6;19:19. doi: 10.1186/s12993-023-00221-y (PMC10626819; doi:10.1186/s12993-023-00221-y)
Supplement: Supplementary file 1 — Additional file 1. Supplementary Information. [file 12993_2023_221_MOESM1_ESM.docx]

**SUPPLEMENTAL INFORMATION**

**RESULTS**

*Whole brain gray matter, white matter, and cerebrospinal fluid*

No significant differences between phenotypes were observed in whole brain volume (total volume in mm^3^: df = 22; T-test = 1.19; p = 0.24), GM (percentage of volume: df = 22; T-test = -0.93; p = 0.36; total volume in mm^3^: df = 22; T-test = -0.86; p = 0.4) or CSF (percentage of volume: df = 22; T-test = -1.11; p = 0.28; total volume in mm^3^: df = 22; T-test = -1.03; p = 0.31). However, T-test analysis revealed that the percentage of WM volume was higher in HD animals compared to LD animals (df = 22; T-test = -2.66; p < 0.05; d= 1.09). This difference was also statistically significant in the total volume (mm^3^) of WM (df = 22; T-test = -2.8; p < 0.05; d= 1.14). Moreover, there was a trend to positive correlation between water consumed during the last five sessions of SIP and volume of WM (in %: r = 0.38; p = 0.06; in mm^3^: r = 0.39; p = 0.06).

*White matter structures*

T-test analysis revealed that HD animals showed an increased volume in the Corpus Callosum (CC) (percentage of volume: df = 22; T-test = -2.95; p < 0.05; d = 1.4; total volume in mm^3^: df = 22; T-test = -3.01; p < 0.05; d = 1.23) and Anterior Commissure (AC) (percentage of volume: df = 22; T-test = -3.1; p < 0.01; d = 1.38; total volume in mm^3^: df = 22; T-test = -3.17; p < 0.01; d = 1.29) compared to LD animals.

*Gray matter structures: cortical areas*

T-test analysis revealed that HD animals showed an increased volume of motor cortex (percentage of volume: df = 22; T-test = -2.72; p < 0.05; d = 1; total volume in mm^3^: df = 22; T-test = -2.52; p < 0.05; d = 1,03) and dlOFC (percentage of volume: df = 22; T-test = -2.19; p < 0.05; d = 0.85; total volume in mm^3^: df = 22; T-test = -2.08; p < 0.05; d = 0.86) compared to LD animals. However, compulsive HD presented a decreased volume of mPFC compared to LD rats (percentage of volume: df = 22; T-test =2.54 ; p < 0.05; d = 1,13; total volume in mm^3^: df = 22; T-test = 2.8; p < 0.05; d = 1,16).

Water consumed (ml) during the last 5 sessions on SIP correlated with volume of motor cortex (in %: r = 0.5; p < 0.05; in mm^3^: r = 0.49; p < 0.05). A trend in correlations between water intake on SIP and volume of mPFC (in mm^3^: r = -0.36; p = 0.08) was also found. Moreover, licking behavior during the last 5 sessions on SIP correlated with volume of Motor Cortex (in %: r = 0.57; p < 0.01; in mm^3^: r = 0.58; p < 0.01) and mPFC (in %: r = -0.47; p < 0.05; in mm^3^: r = 0.49; p < 0.05).

*Gray matter structures: subcortical anterior areas*

T-test analysis revealed that HD animals showed an increased volume in striatum (percentage of volume: df = 22; T-test = -2.44; p < 0.05; d = 1.26; total volume in mm^3^: df = 22; T-test = -2.28; p < 0.05; d = 0.93), and Preoptic Area (POA) (percentage of volume: df = 22; T-test = -2.59; p < 0.05; d = 1,17; total volume in mm^3^: df = 22; T-test = -2.49; p < 0.05; d = 1.02) compared LD rats.

Moreover, water consumed (ml) during the last 5 sessions on SIP correlated with volume of Striatum (in %: r = 0.43; p < 0.05; in mm^3^: r = 0.44; p < 0.05) and POA (in %: r = 0.51; p < 0.01; in mm^3^: r = 0.52; p < 0.01). A trend to correlate between water intake on SIP and volume of POA (in %: r = 0.37; p = 0.76; in mm^3^: r = 0.38; p = 0.06) was also found.

*Gray matter structures: subcortical medial areas*

T-test analysis revealed that HD animals showed increased volume in amygdala (percentage of volume: df = 22; T-test = -3.21; p < 0.01; d = 1.54; total volume in mm^3^: df = 22; T-test = -3.05; p < 0.01; d = 1.24), dentate gyrus (DG) (percentage of volume: df = 22; T-test = -2.72; p < 0.05; d = 1.5; total volume in mm^3^: df = 22; T-test = -2.72; p < 0.05; d = 1.12) and STN (percentage of volume: df = 22; T-test = -2.18; p < 0.05; d = 0.91; total volume in mm^3^: df = 22; T-test = -2.17; p < 0.05; d = 0.88).

Water consumed (ml) during the last 5 sessions on SIP correlated with volume of Amygdala (in %: r = 0.47; p < 0.05; in mm^3^: r = 0.47; p < 0.05). A trend to correlate between water intake on SIP and volume of DG in water intake (in %: r = 0.38; p = 0.07; in mm^3^: r = 0.38; p = 0.07) and licks (in %: r = 0.36; p = 0.08; in mm^3^: r = 0.37; p = 0.07) was also found.

*Gray matter structures: subcortical posterior areas*

T-test analysis revealed that HD animals showed increased volume in periaqueductal gray (PAG) (percentage of volume: df = 22; T-test = -3.2; p < 0.01; d = 2.22; total volume in mm^3^: df = 22; T-test = -3.13; p < 0.01; d = 1.29), midbrain (percentage of volume: df = 22; T-test = -2.46; p < 0.05; d = 0.85; total volume in mm^3^: df = 22; T-test = -2.21; p < 0.05; d = 0.9) and parasubiculum (PaS) (percentage of volume: df = 22; T-test = -2.68; p < 0.05; d = 1,13; total volume in mm^3^: df = 22; T-test = -2.62; p < 0.05; d = 0.7).

Water consumed (ml) during the last 5 sessions on SIP correlated with volume of PAG (in %: r = 0.66; p < 0.001; in mm^3^: r = 0.67; p < 0.001). Moreover, licking behavior during the last 5 sessions on SIP correlated with volume of PAG (in %: r = 0.62; p < 0.001; in mm^3^: r = 0.64; p < 0.001).

*Cerebellum*

T-test analysis revealed that HD animals showed increased volume in Cerebellum compared to LD animals (percentage of volume: df = 22; T-test = -2.37; p < 0.05; d = 0.99; total volume in mm^3^: df = 22; T-test = -2.18; p < 0.05; d = 0.89).

Volumetric MRI data of brain structures are presented in Table S1 and correlations between water intake and the number of licks on SIP and MRI data are shown in Table S2 and S3.

|  | **Volume (mm^3^)** | | **Volume (%)** | |
| --- | --- | --- | --- | --- |
|  | **LD** | **HD** | **LD** | **HD** |
| **Insular Cortex** | 58,97 ± 0,27 | 58,30 ± 0,26 | 0,53 ± 0,002 | 0,53± 0,003 |
| **Auditory Cortex** | 44,64 ± 0,36 | 45,92 ± 0,29 | 0,40 ± 0,004 | 0,42 ± 0,003 |
| **Cingulate Cortex** | 39,72 ± 0,15 | 39,63 ± 0,2 | 0,36 ± 0,001 | 0,36 ± 0,002 |
| **Enthorhinal Cortex** | 33,98 ± 0,25 | 34,67 ± 0,204 | 0,31 ± 0,002 | 0,31 ± 0,002 |
| **Motor Cortex** | 72,93 ± 0,37 | 75,55 ± 0,46* (d=1,03) | 0,66 ± 0,004 | 0,69 ± 0,004*  (d=1) |
| **Parietal Cortex** | 9,75 ± 0,14 | 10,34 ± 0,16 | 0,09 ± 0,001 | 0,09 ± 0,001 |
| **Restrosplenial Cortex** | 34,49 ± 0,2 | 34,20 ± 0,31 | 0,31 ± 0,002 | 0,31 ± 0,003 |
| **Primary somatosensory Cortex** | 136,47 ± 0,79 | 137 ± 0,49 | 1,24 ± 0,008 | 1,24 ± 0,005 |
| **Primary visual Cortex** | 43,51 ± 0,45 | 43,69 ± 0,26 | 0,39 ± 0,004 | 0,4 ± 0,002 |
| **Secondary visual Cortex** | 20,26 ± 0,14 | 19,63 ± 0,19 | 0,18 ± 0,001 | 0,18 ± 0,002 |
| **Ectorhinal Cortex** | 23,61 ± 0,1 | 23,30 ± 0,22 | 0,21 ± 0,001 | 0,21 ± 0,002 |
| **Frontal Cortex, Area 3** | 12,41 ± 0,1 | 12,09 ± 0,06 | 0,11 ± 0,001 | 0,11 ± 0,001 |
| **Lateral parietal association Cortex** | 10,04 ± 0,14 | 10,06 ± 0,16 | 0,09 ± 0,001 | 0,09 ± 0,001 |
| **Perirhinal Cortex** | 0,51 ± 0,02 | 0,48 ± 0,01 | 0,005 ± 0,00001 | 0,004 ± 0,00001 |
| **Secondary somatosensory Cortex** | 9 ± 0,08 | 8,71 ± 0,07 | 0,08 ± 0,001 | 0,08 ± 0,001 |
| **Temporal association Cortex** | 8,04 ± 0,08 | 7,93 ± 0,06 | 0,07 ± 0,001 | 0,07 ± 0,001 |
| **Frontal association Cortex** | 3,60 ± 0,06 | 3,71 ± 0,05 | 0,03 ± 0,001 | 0,03 ± 0,0005 |
| **Medial Prefrontal Cortex** | 20,22 ± 0,15 | 19,33 ± 0,1*  (d=1,16) | 0,18 ± 0,001 | 0,18 ± 0,001* (d=1,13) |
| **Cingulum** | 4,07 ± 0,05 | 4,12 ± 0,03 | 0,04 ± 0,0005 | 0,04 ± 0,0003 |
| **Midbrain** | 99,12 ± 0,763 | 102,78 ± 0,54* (d=0,9) | 0,90 ± 0,007 | 0,93 ± 0,005* (d=0,85) |
| **Septum** | 17,07 ± 0,09 | 17,41 ± 0,07 | 0,15 ± 0,001 | 0,16 ± 0,001 |
| **Diagonal domain** | 5,58 ± 0,05 | 5,61 ± 0,03 | 0,05 ± 0,0004 | 0,05 ± 0,0003 |
| **Hypothalamus** | 43,53 ± 0,23 | 43,56 ± 0,22 | 0,39 ± 0,002 | 0,40 ± 0,002 |
| **Striatum** | 102,10 ± 0,75 | 105,59 ± 0,44* (d=0,93) | 0,93 ± 0,007 | 0,96 ± 0,004* (d=1,26) |
| **Diencephalon** | 96,02 ± 1,02 | 94,41 ± 0,66 | 0,87 ± 0,01 | 0,86 ± 0,006 |
| **Internal capsule** | 30,41 ± 0,58 | 31,69 ± 0,87 | 0,28 ± 0,005 | 0,29 ± 0,008 |
| **Pallidum** | 20,17 ± 0,34 | 21,24 ± 0,27 | 0,18 ± 0,003 | 0,19 ± 0,002 |
| **Accumbens nucleus** | 14,66 ± 0,13 | 14,98 ± 0,11 | 0,13 ± 0,001 | 0,14 ± 0,001 |
| **Fimbria** | 17,09 ± 0,25 | 17,87 ± 0,39 | 0,15 ± 0,002 | 0,16 ± 0,004 |
| **Corpus callosum** | 68,03 ± 0,62 | 71 ± 1,12 | 0,62 ± 0,006 | 0,64 ± 0,01 |
| **Amygdala** | 48,09 ± 0,15 | 49,83 ± 0,29** (d=1,24) | 0,44 ± 0,002 | 0,45 ± 0,003** (d=1,54) |
| **Preoptic area** | 9,68 ± 0,11 | 10,27 ± 0,08* (d=1,02) | 0,09 ± 0,001 | 0,09 ± 0,001*  (d=1,17) |
| **Isocortex** | 65,03 ± 0,43 | 63,71 ± 0,38 | 0,59 ± 0,004 | 0,58 ± 0,004 |
| **Cerebellum** | 345,12 ± 1,79 | 354,18 ± 1,51* (d=0,89) | 3,13 ± 0,02 | 3,22 ± 0,01*  (d=0,99) |
| **Olfactory structures** | 128,4 ± 0,66 | 131,21 ± 0,71 | 1,16 ± 0,007 | 1,19 ± 0,007 |
| **Bed nucleus of the stria terminalis** | 3,85 ± 0,06 | 3,98 ± 0,04 | 0,03 ± 0,001 | 0,04 ± 0,0004 |
| **Pituitary** | 7,47 ± 0,15 | 7,62 ± 0,08 | 0,07 ± 0,001 | 0,07 ± 0,001 |
| **Ventricles** | 4,38 ± 0,07 | 4,26 ± 0,08 | 0,04 ± 0,001 | 0,04 ± 0,001 |
| **Pineal gland** | 0,51 ± 0,03 | 0,50 ± 0,03 | 0,005 ± 0,0003 | 0,005 ± 0,0003 |
| **Basal Forebrain Region** | 78,45 ± 0,66 | 80,08 ± 0,5 | 0,71 ± 0,006 | 0,73 ± 0,005 |
| **Cornu Ammonis 1** | 31,64 ± 0,63 | 32,67 ± 0,59 | 0,29 ± 0,006 | 0,30 ± 0,006 |
| **Cornu Ammonis 2** | 3,15 ± 0,09 | 3,23 ± 0,09 | 0,03 ± 0,001 | 0,03 ± 0,001 |
| **Cornu Ammonis 3** | 25 ± 0,48 | 25,22 ± 0,57 | 0,23 ± 0,004 | 0,23 ± 0,005 |
| **Dentate Gyrus** | 35,86 ± 0,43 | 38,61 ± 0,37* (d=1,12) | 0,32 ± 0,004 | 0,35 ± 0,004* (d=1,5) |
| **Dorso Lateral Orbital Cortex** | 5,80 ± 0,09 | 6,25 ± 0,08* (d=0,86) | 0,05 ± 0,001 | 0,06 ± 0,001* (d=0,85) |
| **Globus Pallidus** | 0,03 ± 0,004 | 0,04 ± 0,004 | 0,0003 ± 0,00003 | 0,0003 ± 0,00003 |
| **Parasubiculum** | 8,58 ± 0,17 | 9,61 ± 0,15*  (d=0,7) | 0,08 ± 0,002 | 0,09 ± 0,001* (d=1,13) |
| **Periaqueductal Gray** | 24,66 ± 0,16 | 25,91 ± 0,15** (d=1,29) | 0,22 ± 0,002 | 0,24 ± 0,002** (d=2,22) |
| **Substantia Nigra** | 3,70 ± 0,06 | 3,95 ± 0,09 | 0,03 ± 0,001 | 0,04 ± 0,001 |
| **Anterior Commissure** | 4,22 ±,034 | 4,51 ± 0,04** (d=1,29) | 0,04 ± 0,0003 | 0,04 ± 0,0004** (d=1,38) |
| **Corpus Callosum and Associated Subcortical White Matter** | 74,47 ± 0,77 | 81,05 ± 0,967 | 0,67 ± 0,007 | 0,74 ± 0,01 |
| **Posterior Commissure** | 0,26 ± 0,03 | 0,21 ± 0,02 | 0,002 ± 0,0003 | 0,002 ± 0,0002 |
| **Pyramidal tract** | 0,04 ± 0,006 | 0,05 ± 0,006 | 0,0004 ± 0,0001 | 0,0004 ± 0,0001 |
| **Spinal Cord** | 4,72 ± 0,39 | 4,64 ± 0,35 | 0,04 ± 0,004 | 0,04 ± 0,003 |
| **Subthalamic Nucleus** | 0,17 ± 0,007 | 0,21 ± 0,008* (d=0,88) | 0,002 ± 0,0001 | 0,002 ± 0,0001* (d=0,91) |
| **Optic Pathways** | 1,90 ± 0,03 | 2,02 ± 0,03 | 0,02 ± 0,0003 | 0,02 ± 0,0003 |
| **Thalamus** | 51,95 ± 1,37 | 53,38 ± 1,44 | 0,47 ± 0,012 | 0,49 ± 0,01 |
| **Ventral Hippocampal Commissure** | 0,78 ± 0,04 | 0,81 ± 0,06 | 0,007 ± 0,0004 | 0,007 ± 0,0005 |
| **Periventricular Grey** | 11,92 ± 0,27 | 11,82 ± 0,28 | 0,11 ± 0,002 | 0,11 ± 0,003 |

**Table 1.** Volumetric MRI data. Data are expressed as the means ± SEM. *p < 0.05; **p < 0.01; ***p < 0.001 indicate significant differences between LD and HD rats.

|  | **Volume (mm^3^) – ml on SIP** | | **Volume (%) – ml on SIP** | |
| --- | --- | --- | --- | --- |
|  | **r-value** | **p-value** | **r-value** | **p-value** |
| **Total volume** | -0,22 | 0,3 |  |  |
| **Grey Matter** | 0,15 | 0,47 | 0,17 | 0,44 |
| **White Matter** | 0,39 | 0,06 (trend) | 0,38 | 0,06 (trend) |
| **Cerebrospinal fluid** | 0,22 | 0,30 | 0,23 | 0,28 |
| **Insular Cortex** | -0,12 | 0,58 | -0,08 | 0,72 |
| **Auditory Cortex** | 0,21 | 0,33 | 0,22 | 0,31 |
| **Cingulate Cortex** | -0,07 | 0,73 | -0,04 | 0,86 |
| **Enthorinal Cortex** | 0,1 | 0,62 | 0,12 | 0,56 |
| **Motor Cortex** | 0,49 | 0,01* | 0,5 | 0,01* |
| **Parietal Cortex** | 0,29 | 0,16 | 0,3 | 0,16 |
| **Restrosplenial Cortex** | -0,25 | 0,23 | -0,22 | 0,29 |
| **Primary somatosensory Cortex** | 0,07 | 0,73 | 0,1 | 0,65 |
| **Primary visual Cortex** | 0,05 | 0,81 | 0,07 | 0,76 |
| **Secondary visual Cortex** | -0,13 | 0,55 | -0,11 | 0,62 |
| **Ectorhinal Cortex** | 0,07 | 0,75 | 0,09 | 0,68 |
| **Frontal Cortex, Area 3** | -0,36 | 0,08 (trend) | -0,33 | 0,12 |
| **Lateral parietal association Cortex** | -0,01 | 0,95 | 0,00 | 0,99 |
| **Perirhinal Cortex** | -0,07 | 0,73 | -0,07 | 0,74 |
| **Secondary somatosensory Cortex** | -0,29 | 0,16 | -0,28 | 0,19 |
| **Temporal association Cortex** | -0,08 | 0,71 | -0,06 | 0,79 |
| **Frontal association Cortex** | 0,21 | 0,33 | 0,22 | 0,3 |
| **Medial Prefrontal Cortex** | -0,36 | 0,08 (trend) | -0,33 | 0,12 |
| **Cingulum** | 0,12 | 0,59 | 0,13 | 0,56 |
| **Midbrain** | 0,27 | 0,21 | 0,29 | 0,17 |
| **Septum** | 0,06 | 0,77 | 0,08 | 0,70 |
| **Diagonal domain** | -0,03 | 0,9 | 0,00 | 0,98 |
| **Hypothalamus** | 0,1 | 0,65 | 0,12 | 0,57 |
| **Striatum** | 0,44 | 0,03* | 0,43 | 0,04* |
| **Diencephalon** | -0,19 | 0,37 | -0,17 | 0,43 |
| **Internal capsule** | 0,13 | 0,55 | 0,13 | 0,53 |
| **Pallidum** | 0,19 | 0,37 | 0,20 | 0,35 |
| **Accumbens nucleus** | 0,21 | 0,33 | 0,23 | 0,29 |
| **Fimbria** | 0,19 | 0,37 | 0,20 | 0,35 |
| **Corpus callosum** | 0,2 | 0,35 | 0,21 | 0,33 |
| **Amygdala** | 0,47 | 0,02* | 0,47 | 0,02* |
| **Preoptic area** | 0,52 | 0,01* | 0,51 | 0,01* |
| **Isocortex** | -0,35 | 0,09 | -0,31 | 0,14 |
| **Cerebellum** | 0,28 | 0,18 | 0,29 | 0,17 |
| **Olfactory structures** | 0,44 | 0,03* | 0,44 | 0,03* |
| **Bed nucleus of the stria terminalis** | 0,3 | 0,15 | 0,31 | 0,15 |
| **Pituitary** | -0,01 | 0,95 | 0,00 | 0,99 |
| **Ventricles** | -0,02 | 0,92 | -0,01 | 0,96 |
| **Pineal gland** | -0,07 | 0,77 | -0,06 | 0,77 |
| **Basal Forebrain Region** | 0,24 | 0,25 | 0,26 | 0,21 |
| **Cornu Ammonis 1** | 0,04 | 0,85 | 0,05 | 0,80 |
| **Cornu Ammonis 2** | 0,03 | 0,90 | 0,04 | 0,86 |
| **Cornu Ammonis 3** | 0,05 | 0,80 | 0,07 | 0,76 |
| **Dentate Gyrus** | 0,38 | 0,07 (trend) | 0,38 | 0,07 (trend) |
| **Dorso Lateral Orbital Cortex** | 0,26 | 0,22 | 0,28 | 0,19 |
| **Globus Pallidus** | 0,10 | 0,64 | 0,11 | 0,63 |
| **Parasubiculum** | 0,27 | 0,21 | 0,28 | 0,19 |
| **Periaqueductal Gray** | 0,67 | 0,0004*** | 0,66 | 0,0004*** |
| **Substantia Nigra** | 0,18 | 0,40 | 0,19 | 0,37 |
| **Anterior Commissure** | 0,93 | 0,23 | 0,94 | 0,21 |
| **Corpus Callosum and Associated Subcortical White Matter** | 0,87 | 0,32 | 0,90 | 0,29 |
| **Posterior Commissure** | 0,17 | 0,89 | 0,18 | 0,89 |
| **Pyramidal tract** | -0,83 | 0,38 | -0,82 | 0,39 |
| **Spinal Cord** | -0,98 | 0,12 | -0,98 | 0,13 |
| **Subthalamic Nucleus** | 0,19 | 0,37 | 0,19 | 0,36 |
| **Optic Pathways** | 0,31 | 0,14 | 0,31 | 0,14 |
| **Thalamus** | 0,72 | 0,49 | 0,75 | 0,46 |
| **Ventral Hippocampal Commissure** | 0,27 | 0,83 | 0,29 | 0,81 |
| **Periventricular Grey** | -0,65 | 0,55 | -0,61 | 0,58 |
| **ventricular system** | 0,89 | 0,30 | 0,93 | 0,25 |

**Table 2.** Correlations between MRI metrics and water intake on SIP. *p < 0.05; **p < 0.01; ***p < 0.001 indicate significant differences between LD and HD rats.

|  | **Volume (mm^3^) – Licks on SIP** | | **Volume (%) – Licks on SIP** | |
| --- | --- | --- | --- | --- |
|  | **r-value** | **p-value** | **r-value** | **p-value** |
| **Total volume** | -0,10 | 0,65 |  |  |
| **Grey Matter** | 0,04 | 0,84 | 0,06 | 0,80 |
| **White Matter** | 0,31 | 0,14 | 0,29 | 0,17 |
| **Cerebrospinal fluid** | 0,15 | 0,47 | 0,16 | 0,46 |
| **Insular Cortex** | -0,29 | 0,17 | -0,32 | 0,13 |
| **Auditory Cortex** | 0,15 | 0,49 | 0,15 | 0,49 |
| **Cingulate Cortex** | -0,06 | 0,79 | -0,07 | 0,73 |
| **Enthorinal Cortex** | 0,02 | 0,94 | 0,01 | 0,98 |
| **Motor Cortex** | 0,58 | 0,003** | 0,57 | 0,004** |
| **Parietal Cortex** | 0,38 | 0,07 (trend) | 0,39 | 0,06 (trend) |
| **Restrosplenial Cortex** | -0,28 | 0,18 | -0,30 | 0,15 |
| **Primary somatosensory Cortex** | 0,08 | 0,72 | 0,07 | 0,73 |
| **Primary visual Cortex** | 0,10 | 0,63 | 0,10 | 0,63 |
| **Secondary visual Cortex** | -0,33 | 0,12 | -0,34 | 0,10 |
| **Ectorhinal Cortex** | -0,13 | 0,55 | -0,14 | 0,51 |
| **Frontal Cortex, Area 3** | -0,27 | 0,21 | -0,28 | 0,18 |
| **Lateral parietal association Cortex** | -0,02 | 0,94 | -0,02 | 0,93 |
| **Perirhinal Cortex** | -0,01 | 0,97 | -0,01 | 0,96 |
| **Secondary somatosensory Cortex** | -0,17 | 0,42 | -0,18 | 0,40 |
| **Temporal association Cortex** | 0,03 | 0,88 | 0,02 | 0,91 |
| **Frontal association Cortex** | 0,11 | 0,62 | 0,10 | 0,64 |
| **Medial Prefrontal Cortex** | -0,49 | 0,02* | -0,47 | 0,02* |
| **Cingulum** | -0,07 | 0,75 | -0,08 | 0,72 |
| **Midbrain** | 0,36 | 0,09 | 0,35 | 0,10 |
| **Septum** | 0,10 | 0,64 | 0,10 | 0,65 |
| **Diagonal domain** | -0,07 | 0,74 | -0,08 | 0,70 |
| **Hypothalamus** | 0,03 | 0,89 | 0,02 | 0,93 |
| **Striatum** | 0,29 | 0,16 | 0,31 | 0,15 |
| **Diencephalon** | -0,03 | 0,88 | -0,04 | 0,86 |
| **Internal capsule** | 0,11 | 0,61 | 0,11 | 0,62 |
| **Pallidum** | -0,01 | 0,97 | -0,01 | 0,95 |
| **Accumbens nucleus** | 0,16 | 0,46 | 0,15 | 0,47 |
| **Fimbria** | 0,09 | 0,66 | 0,09 | 0,67 |
| **Corpus callosum** | 0,24 | 0,27 | 0,24 | 0,27 |
| **Amygdala** | 0,28 | 0,19 | 0,29 | 0,17 |
| **Preoptic area** | 0,38 | 0,06 (trend) | 0,37 | 0,07 (trend) |
| **Isocortex** | -0,43 | 0,04* | -0,4 | 0,05 (trend) |
| **Cerebellum** | 0,22 | 0,31 | 0,22 | 0,29 |
| **Olfactory structures** | 0,21 | 0,32 | 0,21 | 0,32 |
| **Bed nucleus of the stria terminalis** | 0,05 | 0,83 | 0,04 | 0,84 |
| **Pituitary** | 0,17 | 0,41 | 0,18 | 0,41 |
| **Ventricles** | 0,03 | 0,90 | 0,02 | 0,91 |
| **Pineal gland** | -0,04 | 0,84 | -0,05 | 0,83 |
| **Basal Forebrain Region** | 0,05 | 0,82 | 0,06 | 0,77 |
| **Cornu Ammonis 1** | -0,01 | 0,97 | 0,00 | 1,00 |
| **Cornu Ammonis 2** | 0,04 | 0,87 | 0,04 | 0,85 |
| **Cornu Ammonis 3** | -0,05 | 0,80 | -0,04 | 0,84 |
| **Dentate Gyrus** | 0,37 | 0,07 (trend) | 0,36 | 0,08 (trend) |
| **Dorso Lateral Orbital Cortex** | 0,17 | 0,42 | 0,18 | 0,40 |
| **Globus Pallidus** | -0,06 | 0,79 | -0,06 | 0,79 |
| **Parasubiculum** | 0,28 | 0,18 | 0,29 | 0,18 |
| **Periaqueductal Gray** | 0,64 | 0,001*** | 0,62 | 0,001*** |
| **Substantia Nigra** | 0,33 | 0,11 | 0,33 | 0,12 |
| **Anterior Commissure** | 0,91 | 0,27 | 0,92 | 0,25 |
| **Corpus Callosum and Associated Subcortical White Matter** | 0,90 | 0,29 | 0,92 | 0,26 |
| **Posterior Commissure** | 0,12 | 0,92 | 0,12 | 0,92 |
| **Pyramidal tract** | -0,86 | 0,34 | -0,85 | 0,35 |
| **Spinal Cord** | -0,99 | 0,08 | -0,99 | 0,09 |
| **Subthalamic Nucleus** | 0,25 | 0,24 | 0,25 | 0,25 |
| **Optic Pathways** | 0,21 | 0,31 | 0,21 | 0,31 |
| **Thalamus** | 0,76 | 0,45 | 0,78 | 0,43 |
| **Ventral Hippocampal Commissure** | 0,33 | 0,79 | 0,35 | 0,78 |
| **Periventricular Grey** | -0,69 | 0,52 | -0,65 | 0,55 |
| **ventricular system** | 0,86 | 0,34 | 0,9 | 0,28 |

**Table 3.** Correlations between MRI metrics and number of licks on SIP. *p < 0.05; **p < 0.01; ***p < 0.001 indicate significant differences between LD and HD rats.
